# Biochemical features of the novel Tail Tubular Protein A of *Yersinia* phage phiYeO3-12

**DOI:** 10.1038/s41598-020-61145-5

**Published:** 2020-03-06

**Authors:** Anna Pyra, Natalia Urbańska, Karolina Filik, Katherine Tyrlik, Ewa Brzozowska

**Affiliations:** 1 University of Wroclaw, Faculty of Chemistry, 14F. Joliot-Curie St, Wroclaw, 50383 Poland; 2 University of Wroclaw, Faculty of Biological Sciences, Institute of Experimental Biology, 6 Kanonia St, Wroclaw, 50328 Poland; 3 Hirszfeld Institute of Immunology and Experimental Therapy, Polish Academy of Sciences, 12R. Weigl St, Wroclaw, 53114 Poland

**Keywords:** Biochemistry, Computational biology and bioinformatics, Bacteriophages

## Abstract

Tail Tubular Protein A (TTPA) was long thought to be strictly a structural protein of environmental bacteriophages. However, our recent work has suggested that some TTPAs have additional functional features and thus are dual-function proteins. This study introduces a new TTPA family member, TTPAgp11, which belongs to *Yersinia* phage phiYeO3-12. We cloned the gene, expressed it and then purified the phage protein. The protein, including its hydrolytic activity, was characterized. Our enzymatic activity tests showed that TTPAgp11 displayed hydrolytic activity towards Red-starch, suggesting that this enzyme could be classified as part as the α − 1, 4-glucosidase family. Protein folding and aggregation tests indicated that TTPAgp11 is a single-domain protein whose aggregation can be induced by maltose or N-acetylglucosamine. The spatial structure of TTPAgp11 seemed to resemble that of the first reported dual-function TTPA, TTPAgp31, which was isolated from *Klebsiella pneumoniae* phage 32.

## Introduction

Antibiotic-resistant and biofilm-forming bacteria are widespread problems in many aspects of human life, including medicine and food production. A biofilm is a large aggregated structure that is formed when bacteria stick to one another on a solid surface^1^. This adhesion is governed by extracellularly secreted polysaccharides called exopolysaccharides (EPS)^2^. EPS can exist as a shell that is ionically or covalently connected to the cell (capsular EPS) or as a mucus (slime EPS). A promising strategy for eradicating drug-resistant biofilm-forming bacteria is the use of lytic phages that contain enzymes capable of depolymerizing (and thereby destroying) a biofilm^2,3^. Although phages are well known to have antibacterial efficiency^4–8^, they are not commonly used in therapeutic settings. Phages have a high risk of undergoing mutagenesis, resulting in the alteration of their biological nature. Also, our inability to monitor their presence in the body makes phage therapy implication challenging. Obligatory lytic phages are virulent and quickly kill their bacterial hosts by lysing them. A one-time, high dose of bacterial endotoxin can be risky for humans. On the other hand, lysogenic bacteriophages can transfer some virulence factors and thus may be associated with pathogenicity, in a process called “lysogenic conversion”; this is very common in *Escherichia coli, Streptococcus pyogenes, Salmonella enterica*, and *S. aureus*. Prophages, for example, can encode exotoxins, such as those causing the major pathogenicity of *E. coli* EHEC, by inter-prophage interaction (verocytoxins or *Shiga*-toxins) or by *Vibrio cholerae* (A-B-type exotoxin mediated by prophage CTX). In addition, a rather broad spectrum of other proteins play a significant role in bacterial virulence^9^.

Macromolecules, such as proteins, offer more control opportunities, beginning with their production process and ending with the relevant therapeutic effects. Creating a treatment plan using a bacteriophage “cocktail” is not trivial, since several parameters of phage biology have to be considered. First, the pharmaceutical phage production process must be strictly defined and accepted by national authorities including the European Medicines Agency (EMA) in Europe. Then a suitable phage treatment must be individualized for each patient. This means modifying the application frequency, duration of therapy, dosage, and pharmaceutical form for each clinical trial or individual therapy^10^.

Tail tubular protein A (TTPA) has been described as a structural protein^11^. TTPA is also called the gatekeeper protein, since it forms the attachment for the tail spikes and is also thought to mediate the initiation of infection through sensing the deflection of the side fibers upon cell wall binding. TTPA, during infection of bacteria, interacts with the side fibers and constitutes the conformational switch by which the side fibers engage causing the release of the capsid contents. TTPA remains in contact with tubular tail protein B (TTPB) which forms a nozzle-like structure mounted below TTPA and is thought to extend the tube through which the DNA travels. In contrast to TTPA, TTPB seems more like an adapter for mounting additional functions than an essential component of the virion^12^.

Most phage depolymerases are encoded in phage structural proteins such as with tail fibers, baseplates and neck structures^13^. Thus, these proteins were long thought to function only as structural proteins. However, as previously reported by our group, the TTPAs from *Klebsiella* phages KP32 and KP34 (TTPAgp31 and TTPAgp44, respectively) have biological activity: TTPAgp31 acts as α − 1, 4- glucosidase (EC 3.2.1.20), while TTPAgp44 acts as a α − 1, 1- trehalase (EC 3.2.1.28)^14,15^. Due to the structural and hydrolytic features of both TTPAs, they were designated as dual-function proteins. The crystal structure of TTPAgp31 (PDB code: 5 mu4) was also described, and it appeared that the protein contained 3D structural elements that distinguished it from all others found in the Protein Databank (PDB)^14^. It was hypothesized that the lytic properties of these TTPAs arised from the presence of an additional structural element that is not found in the other TTPAs reported to date.

TTPA has also been found among the structural proteins of *Yersinia enterocolitica* bacteriophage phiYeO3-12^16^. *Yersinia enterocolitica* is a Gram-negative bacterium that causes food-borne acute or chronic gastrointestinal diseases^17^. The identified phage infects pathogenic strains of *Yersinia enterocolitica* with serotypes O:3 by recognizing bacterial lipopolysaccharides^18^.

Here, is the first report regarding the biochemical properties of the TTPA, *Yersinia* TTPAgp11. This dual-function TTPA acts as a hydrolytic enzyme and is structurally similar to TTPAgp31, which was previously described by our group^14,15^.

## Results and Discussion

The enzymatic activities of the dual-function TTPAs are only just beginning to be studied. As previously reported, the TTPAs encoded by genes 31 and 44 of the *Klebsiella* phages(KP32 and KP34) can hydrolyze saccharide substrates (e.g., maltose, trehalose and starch) as well as bacterial EPS^14,15^. TTPAgp31 resembles maltase in its substrate specificity but lacks a catalytic domain homologous to that of maltase, whereas TTPAgp44 shows trehalase-like activity (UniProt code: D1L2Y9). The question was whether any other TTPAs from bacteriophages could potentially act against biofilm-forming bacteria. After searching the UniProt database^19^ some interesting candidates appeared. From these, the uncharacterized TTPA (UniProt code: Q9T106) encoded by gene 11 of *Yersinia* phage phiYeO3-12 was selected (GenBank code: AJ251805). BLAST amino acid sequence analysis^20^ showed that TTPAgp11 is similar to the TTPAs of phages that infect other types of bacteria with the highest similarity to bacteriophages infecting *Citrobacter* and *Enterobacter (99-100%)* (Supplementary Data). Amino acid sequence similarity was also found between TTPAgp11 and tail fiber protein B from phages of *Yersinia, E. coli* and *Stenotrophomonas* (76–81%). The amino acid sequence diversity found in the TTPAs is likely a reflection of the adaptations that phages acquire in response to environmental changes and/or bacterial cell variability.

**Figure 1:**
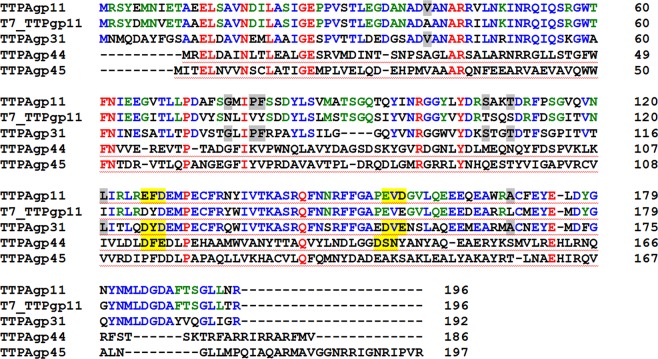
Sequence alignment of TTPAgp11, TTPAgp31 from the tail of bacteriophage KP32 (PDB code: 5 mu4), T7_TTPgp11 from bacteriophage T7 (PDB code: 3j4b), TTPAgp44 from bacteriophage KP34 (UniProt code: D1L2Y9) and TTPAgp45 from *Yersinia* phage phi80–18 (UniProt code: I7K3G0). Identities between TTPAgp11 and all other selected proteins are shown in red; those between TTPAgp11, T7_TTPgp11 and TTPAgp31 are shown in blue; those between TTPAgp11 and T7_TTPgp11 are shown in green, and those between TTPAgp11 and TTPAgp31 are gray-shadowed. For clarity, the identities between TTPAgp11 and TTPgp44 (21%) or TTPAgp45 (20%) are not shown. The catalytic motif proposed herein is shown in yellow.

Previous studies have shown that some tail tubular proteins have hydrolytic activity^14,15^. For this study, our goal was to determine how similar TTPAgp11 is to other proteins in terms of primary structure, since primary structures affect the biological function of proteins. The Clustal Omega tool^21^ was used to perform multiple protein sequence alignments (Fig. 1). For this analysis, two proteins previously reported as enzymes, TTPAgp31 and TTPAgp44^14,15^ were selected. In addition, TTPAgp45, originating from *Yersinia* phage phi80–18^22^, and T7_TTPgp11, which was classified as a tail tubular protein of *Enterobacteria* phage T7 were also chosen. Both of the structures and functions of these proteins have been well documented^11^. Our sequence alignment analysis generated interesting results: TTPAgp11 was found to be most similar to T7_TTPgp11 of phage T7 (80% identity) and TTPAgp31 of phage KP32 (58% identity), whereas it showed far less similarity to TTPAgp44 of phage KP34 (21%) and TTPAgp45 of *Yersinia* phage phi80–18 (20%). It is particularly interesting that the last protein listed, TTPAgp45, showed the lowest similarity with TTPAgp11, although both proteins originated from *Yersinia* phages. If it is assumed that the function of a protein is determined by its primary structure, then it is expected that the biochemical features of TTPAgp11 should be more similar to those of T7_TTPgp11 (from T7 bacteriophage) than those of TTPAgp31. Notably, TTPAgp31 was 64% identical to T7_TTPgp11, but only the former has been reported to exhibit hydrolytic activity^14,15^.

Our Phyre server analysis^23^ showed that 92% and 93% of the amino acids of TTPAgp11 took on the same predicted structures as those of TTPAgp31 and T7_TTPgp11, respectively. The confidence level was 100% for each of these matches. Although the confidence level does not specifically speak to the accuracy of the model, this high level of confidence showed that TTPAgp11 adopted the overall folding and protein core of our models. Any difference in the 3D structure was likely due to deviation of the surface loops relative to those placed in the templates (PDB code: 5 mu4)^14^.

**Figure 2:**
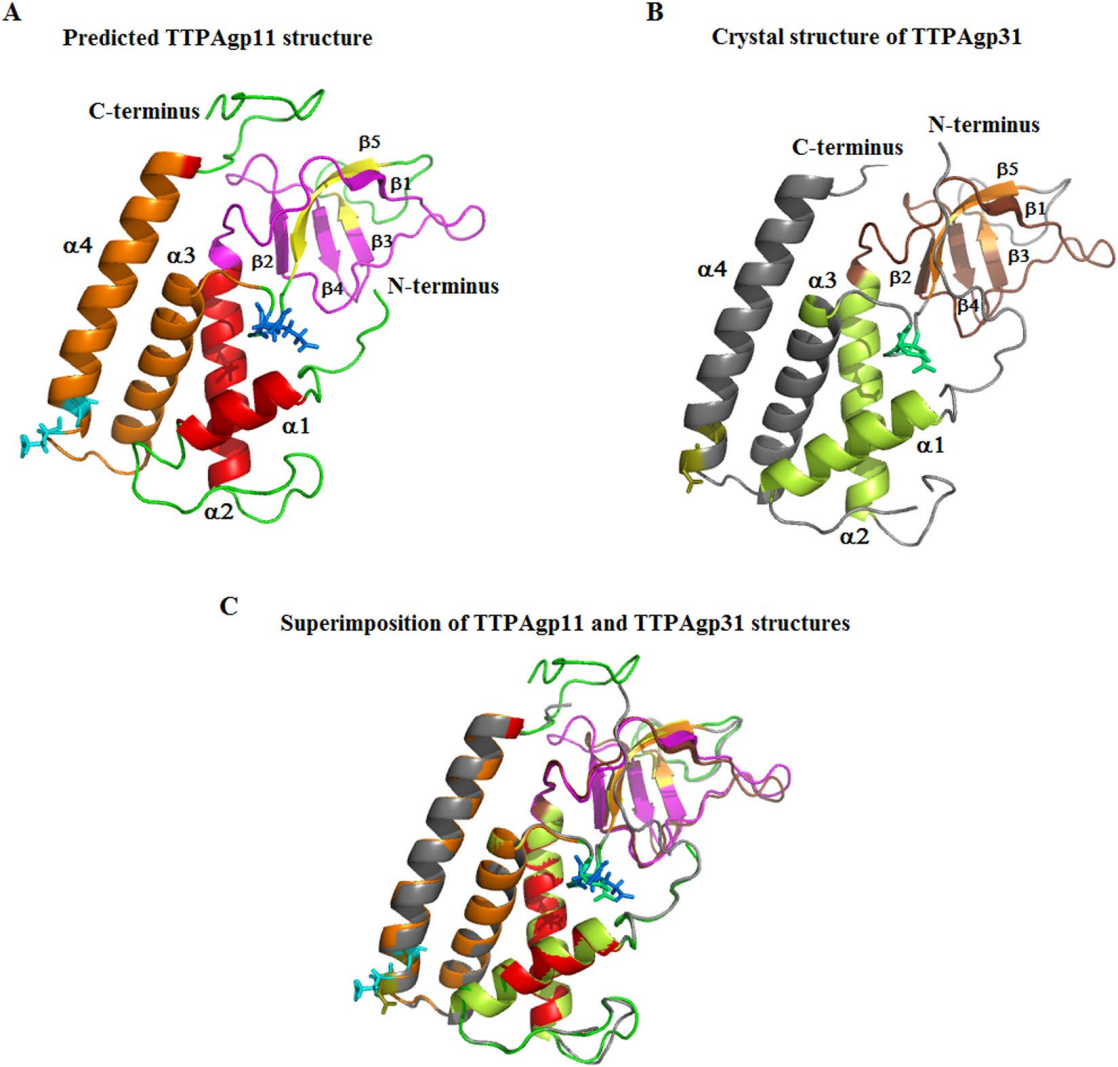
The predicted 3D structural model of TTPAgp11 generated using the I-Tasser server^24,25^. (**A**) The presented structure was the best calculated model, and was obtained using the crystal structure of TTPAgp31 (**B**) deposited in PDB (PDB code: 5 mu4). The residues of two potential catalytic triads are shown as blue and cyan sticks in TTPAgp11 (E126-X-D128 or E155-X-D157) along with green and deepolive sticks (D122-X-D124 or D151-X-E153) in TTPAgp31. A predicted hydrolase domain is shown in orange in TTPAgp11 and in grey in TTPAgp31. A potential lectin-like domain is shown in magenta and brown in TTPAgp11 and TTPAgp31 respectively. (**C**) Predicted structure of TTPAgp11 superimposed onto crystal structure of the TTPAgp31 monomer.

The I-Tasser server^24,25^ was used to further investigate the 3D structure of TTPAgp11. I-Tasser generated five models based on the comparison to the protein structures deposited in PDB (Fig. 2). Interestingly, the server used the structure of TTPAgp31 (PDB code: 5 mu4)^14^, not T7_TTPgp11 (PDB code: 3j4b)^11^, as template, suggesting that the spatial structure was more similar to that of TTPAgp31 versus T7_TTPgp11. In this prediction, the α-helical and β-stranded elements matched well between TTPAgp11 and TTPAgp31, while some differences were seen in the loop regions. For example, the predicted loop containing amino acid residues number 88 to 96 of TTPAgp11 was a bit longer than the corresponding loop of TTPAgp31 (residues 86 to 91). The structural variations between the two models were largely related to the N- and C-termini of the polypeptide chains, but for different reasons. The N-terminal region of TTPAgp31 was nine amino acids longer than that of TTPAgp11, whereas *the* C-terminus of TTPAgp31 is shorter as in the structure determined by X-ray diffraction data the C-terminus could not be modeled as there was no electron density in this region.

Further analysis was performed using the HHPred tool^26^ and it was predicted that TTPAgp11 contained a peptidoglycan hydrolase domain (probability, 68%). Based on our analyses of the primary structure, it was hypothesized that TTPAgp11 could contain a domain in the α-helical region, at amino acid residues 130 to 177, which corresponded to the hydrolase domain of TTPAgp31^14^.

The catalytic motif of most glycosyl hydrolases contained two carboxylates in a D-X-E motif^27^. This short sequence in two regions of TTPAgp31 was previously identified, and it was proposed that one of them is likely to play a crucial role in the enzymatic reaction catalyzed by this protein^14^. Here, two similar and corresponding motifs were found in TTPAgp11 (Fig. 1), but note that they are inverted to the E-X-D sequence (E126-X-D128, E155-X-D157. This suggested, with a high probability, that this motif could be responsible for binding as well as for catalysis of sugar hydrolysis. In our previous studies, in case of TTPAgp31 of the KP32 bacteriophage, it was proposed that D131 and/or D133 were the most likely catalytic amino acid residues. *In silico* analysis presented by Świętnicki & Brzozowska^28^ confirmed that D133 participates in a stable substrate binding. D133 is positioned near a scissile bond, potentially making it a catalytic residue. Our initial *in silico* studies (data not shown) of the putative catalytic mechanism of TTPAgp11 showed that a good catalytic candidate is the E126 residue. In the previously mentioned studies^28^, the authors also confirmed the conformational fit of the saccharide substrate in the catalytic pocket of the protein. Our analysis showed that the catalytic pocket in TTPAgp11 is structurally similar to the catalytic pocket in TTPAgp31. Therefore, it is thought that there is a great probability that, in TTPAgp31, and TTPAgp11, maltose spatially fits into the substrate binding pocket.

It was observed that TTPAgp11, just like TTPAgp31, possessed an additional antiparallel β-sheet carrying a lectin-like domain (residues 56 to 103), which was responsible for sugar binding. The amino acid sequences of these domains in TTPAgp11 and in TTPAgp31 showed 62% identity (Fig. 1).

**Figure 3:**
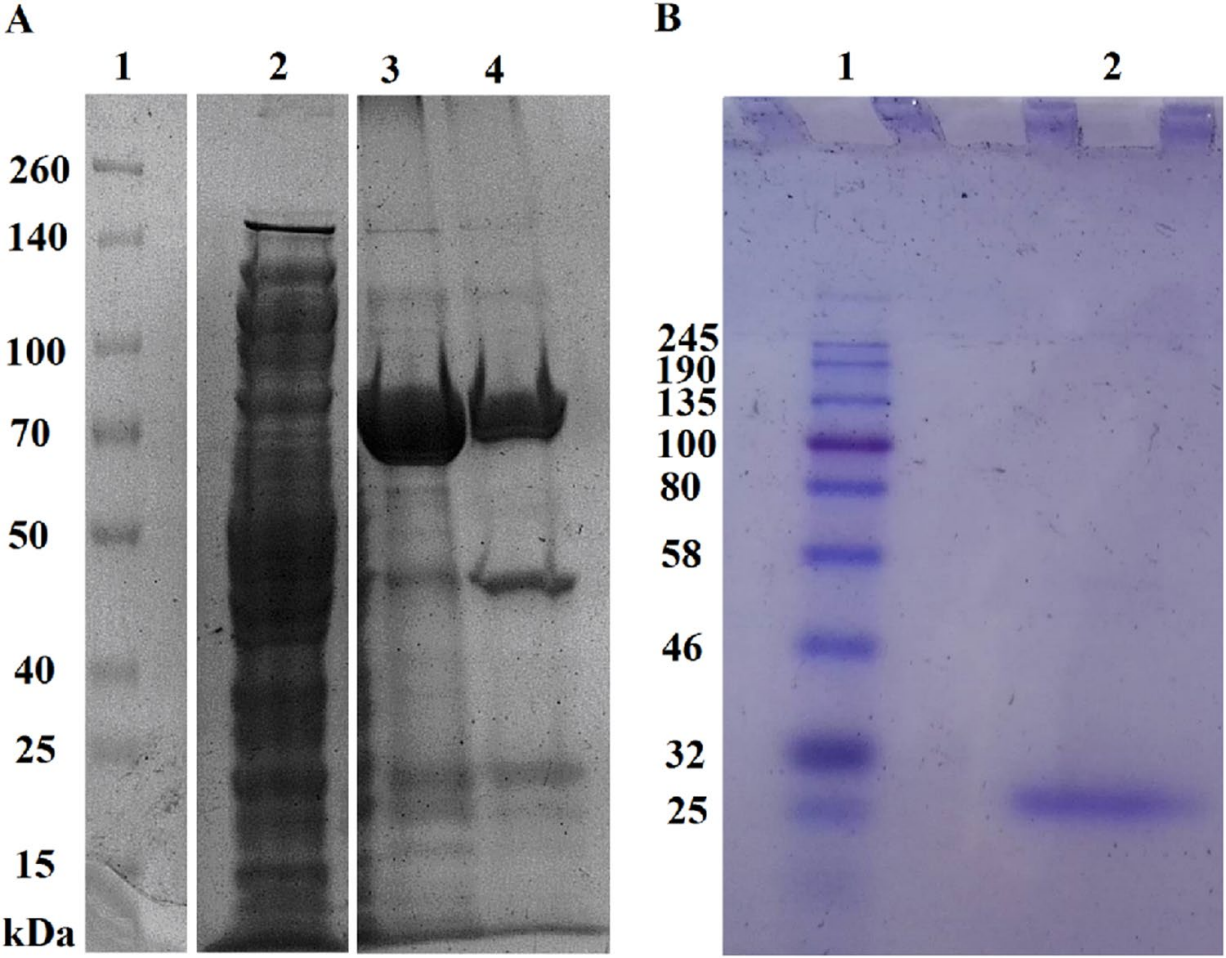
Analysis of TTPAgp11 from *Yersinia* phage phiYeO3-12 using 10% (**A**) and 12.5% (**B**) SDS-PAGE. (**A**) The lanes are as follows: (1) Spectra Multicolor Broad Range Protein Ladder (Bio-Rad); (2) crude extract; (3) pooled fractions after the first round of Ni2+ -affinity chromatography; (4) protein solution after TEV protease cleavage. (**B**) The lanes are as follows: (1) Color Prestained Protein Standard, Broad Range (Bio-Rad); (2) pure target protein - TTPAgp11. The original pictures of the gels are attached in a Supplementary Data File.

**Figure 4:**
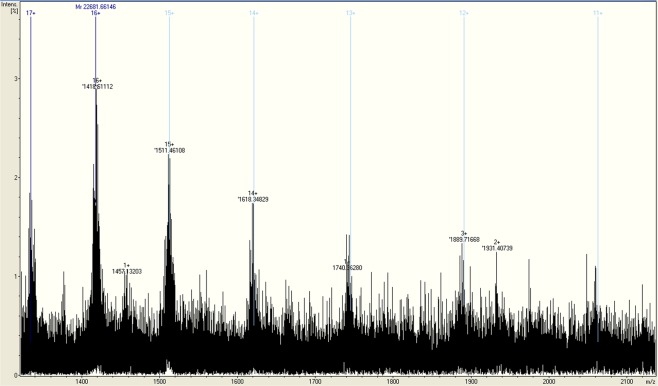
The ESI-MS spectrum of TTPAgp11.

**Figure 5:**
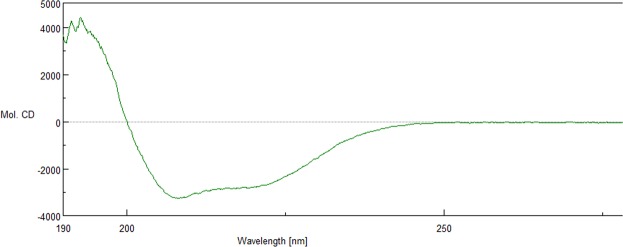
The CD spectrum of TTPAgp11 in units of molar elipticity [deg cm^2^ dmol^−1^].

The gene for TTPAgp11 was cloned into the pMCSG9 vector^29^. Afterwards, the overexpressed protein was purified and analyzed with SDS-PAGE (Fig. 3), electrospray ionization mass spectrometry (ESI-MS), as well as circular dichroism spectroscopy (CD). The obtained ESI-MS spectrum indicated that the apparent molecular weight of this protein was 22.69 kDa (Fig. 4). The results of the CD spectrum analysis (Fig. 5) suggested that TTPAgP11 adopts a mixed α/β secondary structure with a large proportion of helical structure.

**Figure 6:**
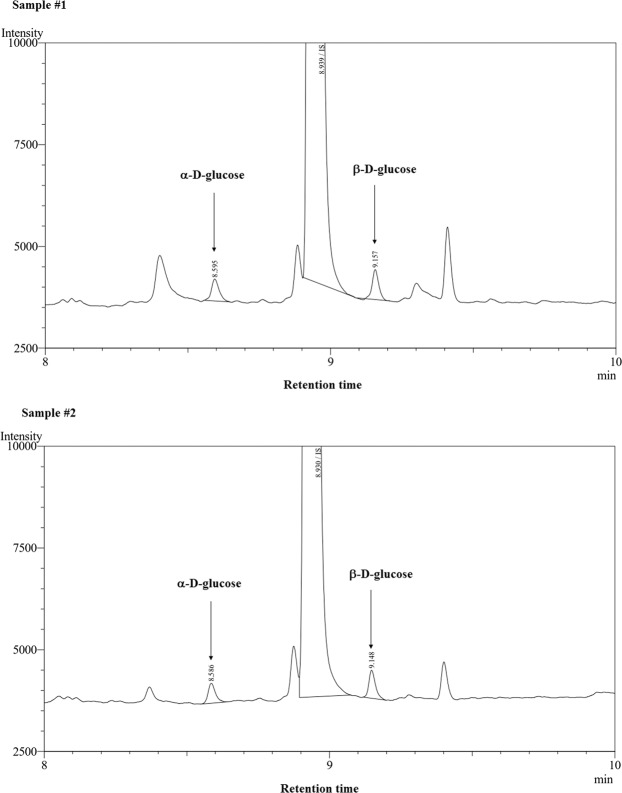
The GC analysis of hydrolytic activity of TTPAgp11 towards maltose. The experiment was performed twice, so two chromatograms are presented. The products of enzymatic reaction are released glucose molecules indicated by the arrows (peaks of α-D-glucose and β-D-glucose). The retention times of the standards of α-D-glucose and β-D-glucose were assigned as 8.599 min and 9.161 min, respectively.

To test the hydrolytic activity of TTAPgp11, disaccharides substrates such as α-lactose, β-lactose, trehalose, melibiose, cellobiose, maltose and saccharose were first used. Then a chromogenic substrate, Red-starch, which was previously found to be hydrolyzed by TTPAgp31, was also used^14^. The hydrolytic activity tests for disaccharides were performed according to the same procedure described in^15^. Our observation was that only maltose was hydrolyzed, however, our results were not unambiguous. To verify the hydrolytic activity of TTPAgp11 towards maltose, gas chromatography of two samples after hydrolysis of this disaccharide by TTPAgp11 was performed. The retention times of standards of α-D-glucose and β-D-glucose were assigned as 8.599 min and 9.161 min, respectively. The GC analysis of the tested samples revealed the presence of α- and β-glucose in both samples (Fig. 6) which were absent in negative control. On the base of the sugars peak sizes it was estimated that around 15–18% of maltose was hydrolyzed. This result undoubtedly indicated hydrolytic activity of TTPAgp11 towards maltose.

**Figure 7:**
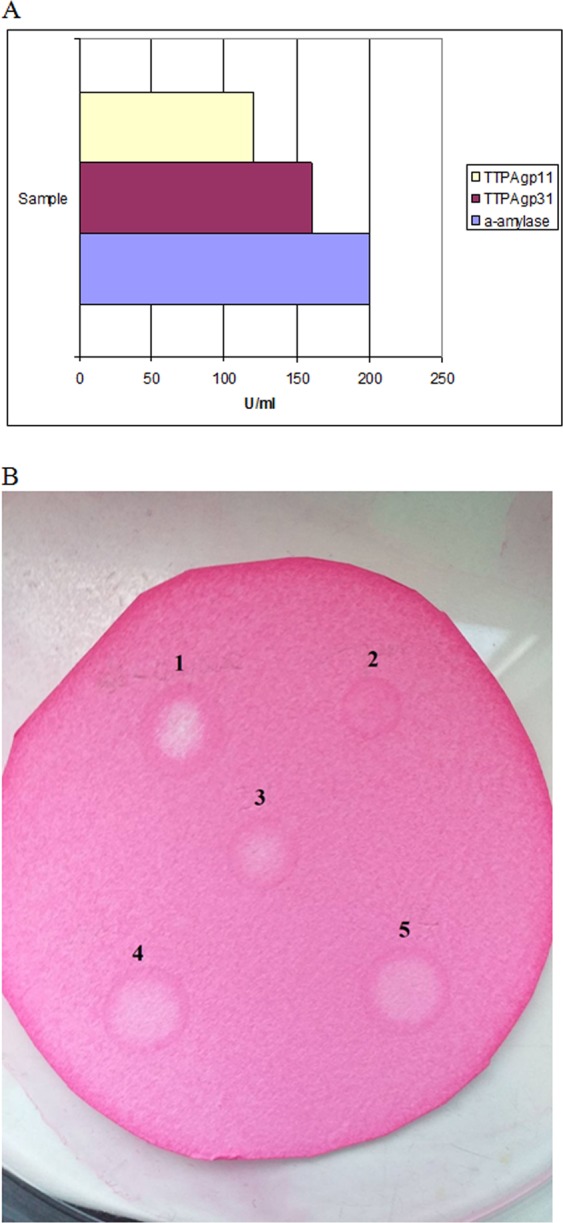
Hydrolytic activity of TTPAgp11 towards Red-starch. *Bacillus subtilis* α-amylase was used as a positive control. (**A**) The α-amylase activity against Red-starch was calculated as described in the provided protocol (Megazyme). All results are presented as averages of results from three independent replicates in three parallel trials. (**B**) Assays were also performed using Red-starch-saturated filter paper treated with: (1) *B. subtilis* α-amylase, as a positive control; (2) phosphate buffer pH 6.8, as a negative control; (3) TTPAgp31 as a positive control; and (4, 5) TTPAgp11.

In order to confirm the activity, another substrate was used. The relevant enzymatic activity was examined both in solution and on filter paper. Our results confirmed that TTPAgp11 hydrolyzed the glycosidic bonds α − 1, 4 in Red-starch to release glucose molecules from the non-reducing end of the substrate (Fig. 7). These findings indicated that, similar to TTPAgp31^14,15^, TTPAgp11 exhibited α-glucosidase-like activity and can be considered to act as an α − 1, 4-glucosidase (EC 3.2.1.20). This further suggested that TTPAgp11 could serve as an antibiofilm factor that acts against the EPS of pathogenic bacteria. In general, this class of enzymes is very diverse in their substrate specificity, optimal reaction temperature and transglucosylation activities^30^.

**Figure 8:**
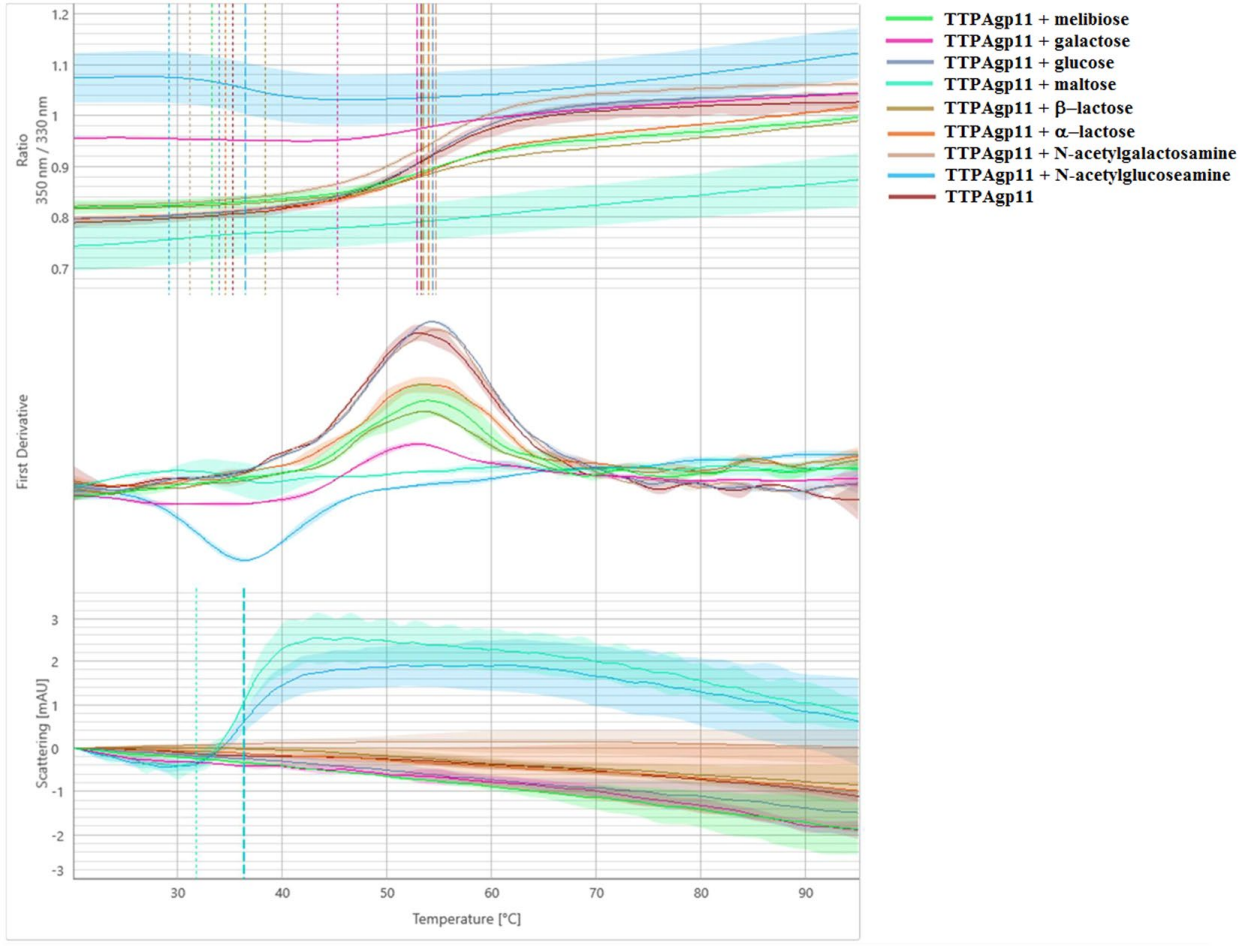
Melting analysis of TTPAgp11 with various sugars. The fluorescence ratio (350 nm/330 nm) is shown in the top panel, the first derivative is shown in the middle panel and scattering is shown in the bottom panel. Thermal unfolding and aggregation onsets (indicated as the vertical dotted lines) as well as unfolding and aggregation transitions (indicated as the vertical dashed lines) are indicated by vertical lines in the graph.

To quantify the stability of TTPAgp11, thermal unfolding and protein aggregation studies were performed using NanoDSF and backreflection technology. The stabilization test was performed in the presence of different monosaccharides (that we considered as potential binders). Moreover, the assessment of protein stability was key to determining the importance for protein’s basic features characterization. Therefore our goal was to characterize the thermal and colloidal stability of TTPAgp11 along with establishing the optimal conditions for large-scale production and long-term storage. The obtained results (Fig. 8, Table 1) showed that TTPAgp11 exhibited one unfolding event at ~53 °C. This suggested that TTPAgp11 is a single-domain protein that can unfold at this temperature. It was further observed that different sugars have different effects on protein stability. The addition, some of them even caused aggregation of TTPAgp11. Aggregation was induced by maltose and N-acetylglucosamine (GlcNAc) but not melibiose, galactose, glucose, lactose or N-acetylgalactosamine (GalNAc). The presence of GlcNAc, but not the other sugars, was associated with an inversion of the unfolding profile. This suggested that this sugar may have a dramatic influence on the conformation of TTPAgp11. These sugar moieties were chosen because they are components of *Y. enterocolitica* lipopolysaccharide. The outer core of the lipopolysaccharide contains two GalNAc moieties, glucose and galactose^18^. Maltose and melibiose were also tested as potential substrates for TTPAgp11.

**Table 1:** TTPAgp11 thermal unfolding and aggregation transitions midpoints (Tm [°C]) obtained from a melting scan of TTPAgp11 mixed with sugars, showing the fluorescence ratio (350 nm/330 nm), the first derivative onset and scattering.

Sample	Tm		
	Fluorescence ratio 350 nm/330 nm	First derivative	Scattering
	[°C]	[°C]	[°C]
TTPAgp11/melibiose	53.93	32.22	NA
TTPAgp11/galactose	52.85	45.25	NA
TTPAgp11/glucose	54.31	33.97	NA
TTPAgp11/maltose	NA	NA	36.26
TTPAgp11/®-lactose	53.44	38.34	NA
TTPAgp11/α-lactose	53.93	34.55	NA
TTPAgp11/N-acetylgalactosamine	54.68	31.19	NA
TTPAgp11/N-acetylglucosamine	36.41	29.11	36.38
TTPAgp11	53.27	35.23	NA

Each sample contained 0.25 mg/ml of TTPAgp11 and 0.15 mg/ml of the sugar.

Glycoside hydrolases catalyze the cleavage of the glycosidic bonds using two commonly found mechanisms of action with either net retention or inversion of anomeric configuration. Hydrolysis, with net retention of anomeric configuration, is achieved via a two steps double displacement mechanism involving a covalent glycosyl-enzyme intermediate. The reaction occurs with acid/base and nucleophilic assistance provided by two amino acid side chains, typically glutamate or aspartate. The second type of hydrolysis - the inversion of anomeric configuration – is achieved via a one step, single-displacement mechanism. The reaction typically occurs with general acid and general base assistance from two amino acid chains usually glutamic or aspartic acids. The distance between both catalytic residues (general acid and general base) is very well conserved, being 5.5 Å and 10.5 Å in retaining and inverting glycoside hydrolases respectively^31^. In the presented studies, two catalytic pairs have been proposed in TTPAgp11. The first pair is E126 – X – D128 and the second one is E155 –X – D157. In the first pair, the distance between acid/base residues is about 6 Å which indicates the retention configuration, while the distance between acid/base in the second pair is over 10 Å indicating the inversion configuration. However, the distances between the putative catalytic residues have been estimated using the predicted 3D structure of the protein (The I-Tasser server). Therefore, the more likely catalytic mechanism of action is retention due to the known proximity of acid/base residues. Moreover, the same configuration was also proposed by Świetnicki & Brzozowska^28^ for the homologous protein possessing the same enzymatic activity. Their conclusion was supported by the results of MD simulations in that work.

Further structural and functional studies are needed to validate the enzymatic function of one or both of these motifs.

## Conclusion

TTPAs were long thought to function solely as structural proteins of bacteriophage tails. However recent studies have shown that they can display hydrolytic activity towards saccharides, and thus act as dual-function proteins. Although some studies have examined the biochemical features of TTPAs, these macromolecules are still not well understood, especially in the context of their enzymatic activity and 3D structure. Here, the goal was to identify a previously uncharacterized TTPA of a new origin, as well as assess its biological function and structure. This research has practical relevance because TTPAs could potentially serve as antimicrobial factors for the treatment of antibiotic-resistant bacterial infection.

This study identified a novel TTPA from *Yersinia* phage phiYeO3-12, and designated it as TTPAgp11. This protein was purified for the first time via a ligation-independent cloning procedure in which gene overexpression was performed in competent *E. coli* cells and the protein was purified by nickel-affinity chromatography. Our hydrolytic activity tests showed that TTPAgp11 can hydrolyze maltose and Red-starch, indicating that it functions as an α-glucosidase. The protein was further classified as part of the α − 1, 4-glucosidase family (EC 3.2.1.20), which also contains the previously reported TTPAgp31. These findings indicated that TTPAgp11 could act against the EPS of biofilm-forming pathogenic bacteria. Our thermal unfolding measurements suggested that TTPAgp11 is a single-domain protein that may unfold around 53 °C and it appeared to aggregate in the presence of maltose or N-acetylglucosamine. Our primary structure analysis revealed high levels of amino acid sequence identity between TTPAgp11 and TTPAgp31 from phage KP32 (58%) and T7_TTPgp11 from phage T7 (80%). Potential catalytic motifs consisting of E-X-D residues were identified and it was proposed that one or both could play a role in the enzymatic reaction. The 3D structure analysis predicted that the spatial structure of TTPAgp11 would be more similar to that of TTPAgp31 than that of T7_TTPgp11. It was speculated that this may reflect the presence of additional structural elements in the crystal structure of TTPAgp31 compared to that of T7_TTPgp11. These elements are likely to be responsible for the hydrolytic activity of the dual-function TTPAs. Clearly, more structural studies are needed to fully clarify the structure and enzymatic reaction center(s) of TTPAgp11.

## Materials and Methods

### Gene cloning and protein overexpression, purification and analysis

The genome of *Yersinia* phage phiYeO3-12 can be found in GenBank under accession number AJ251805. The gp11, the gene that encodes the TTPA studied, was amplified by polymerase chain reaction (PCR) using the following primers: TTPAgp11_FW, TACTTCCAATCCAATGCCATGCGCTCTTATGAGATGAAC and TTPAgp11_RV, TTATCCACTTCCAATGTTATTAGCGGTTAAGTAGACCAGAGG. The PCR reaction was performed using genomic DNA as a template (100 ng). The DNA was isolated from phage lysate using viral DNA extraction kit (Biocompare). The reaction comprised of a 3-min denaturation at 94 °C followed by 35 cycles of 30 s at 94 °C, 45 s at 55–62 °C, and 1 min at 72 °C, and a final extension for 10 min 72 °C. The PCR product was cloned into the pMCSG9 vector using a T4 DNA polymerase, according to the previously described procedure for ligation-independent cloning^29^. The construct was transformed into *E. coli* DH5α cells using the heat-shock method, and confirmed by sequencing. The plasmid was transformed into competent *E.coli* BL21 Star(DE3) cells, which were grown to an OD600 of 0.9 and then induced overnight with 0.25 mM IPTG at 19 °C in Luria-Bertani (LB) medium with ampicillin. The cells were harvested by centrifugation and sonicated 10 times (30-sec pulses separated by 15-sec breaks) in 50 mM Tris/HCl buffer, pH 8.0, containing 300 mM NaCl, 5% glycerol and 5 mM β-mercaptoethanol (buffer A). The cell disruption by sonication was performed on ice (30 s/30 s cycles, 40% amplitude) using a UP200S ultrasonic disintegrator (Dr. Hielscher GmbH).The cell debris was pelleted and the supernatant was loaded onto a nickel-immobilized affinity column equilibrated with buffer A. Unbound proteins were washed with this buffer and TTPAgp11 was eluted with buffer A containing 250 mM imidazole. The tail protein A fractions were pooled together, diluted to a final concentration of 125 mM imidazole and then mixed with TEV protease at a 1:100 ratio with the addition of 0.5 mM EDTA. This mixture was incubated for 2 hr at 30 °C and then overnight at 4 °C to allow cleavage of the MBP-TTPAgp11 fusion protein. The digested proteins were then precipitated with ammonium sulfate (95% saturation) and pelleted to remove the EDTA and imidazole. The proteins were dissolved in buffer A and applied onto a second nickel column. The TTPAgp11-containing His_6_-tag-MBP flow through fractions were collected, precipitated by ammonium sulfate (95% saturation) and analyzed by 10% and 12.5% SDS-PAGE^32^, electrospray ionization mass spectrometry (ESI-MS) and circular dichroism (CD) spectroscopy. Both, for ESI-MS and CD analysis, the protein was dissolved in water with a final concentration of 24 µM. ESI-MS, and measurement was carried out using Bruker micrOTOF-Q mass spectrometer (Bruker Daltonics). The solution of methanol with a 0.1% acetic acid was added to the protein sample in the ratio of 1:1 (v/v) and the mixture was mechanically applied to “electrospray” at 120 ml/min and a 4.5 kV needle voltage. After measurement the molecular mass of the protein was determined using Bruker software^33^. CD measurements were performed four times in a 1 mm cuvette for a one TTPAgp11 sample in the wavelength range of 350 to 190 nm using J-1000 Circular Dichroism spectrophotometer (Jasco Inc.). The results of the CD measurement were given both in units of absorbance and ellipticity. To interpret the obtained spectra, data with the units of ellipticity (millidegrees, mdeg) were used. These results were exported to a spreadsheet and then the values of millidegrees were extracted for 51 points in the wavelength range of 190–240 nm (every 1 nm) and these data were interpreted using K2D3 method that allows to estimate the secondary structure of the protein^34^. The protein concentration was determined by the BCA method^35^.

### Measurement of hydrolytic activity towards a chromogenic substrate and maltose

As a chromogenic substrate for TTPAgp11, 2% Red-starch (Megazyme) was used and prepared in 5 mM KCl. TTPAgp11 (100 µl of 47 µM) was mixed with the substrate (25 µl), water was added to a final volume of 1.5 ml and the sample was incubated at 37 °C for 20 hr. The reaction was stopped using 96% ethanol (2.5 ml). As positive controls, 50 µl of 50 µM α-amylase from *Bacillus subtilis* (Sigma-Aldrich) was used and TTPAgp31 prepared as described for TTPAgp11. After the reactions, the solution’s absorbance at 510 nm were measured. The activity calculations were performed based on a protocol for assay of α-amylase using Red-starch from Megazyme.

The assay was also performed on grade 1 Whatman filter paper, as previously described by Martin *et al*.^36^. Briefly, the filter paper was saturated with 0.6% Red-starch in 50 mM phosphate buffer, pH 6.8, dried, and then spotted with 15 µl of TTPAgp11 at 47 µM, 15 µl of TTPAgp31 at 47 µM (positive control), 10 µl of α-amylase from *Bacillus subtilis* (positive control) and 10 µl of 50 mM phosphate buffer (negative control). The filter paper was incubated at 37 °C for 20 min. Positive results were characterized by a marked color change from dark pink to white.

The hydrolytic activity of TTPAgp11 has been tested towards the disaccharide substrate such as maltose. TTPAgp11 (20 µl of 47 µM) was mixed with the maltose solution (100 µl, 1 mg/ml) prepared in PBS buffer. This buffer was added to a final volume of 0.2 ml and the sample was incubated at room temperature for 24 hr. The test was performed in duplicate. The negative control did not contain the protein. Then the sample was lyophilized and chromatographed in GC-2010 Plus system (Shimadzu, Japan). The sample was derivatized as follows: the dry mass was dissolved in 500 μl of anhydrous pyridine and reacted with 500 μl MTBSTFA (N-tert-Butyldimethylsilyl-N-methyltrifluoroacetamide) as well as 50 μl TMCS (Trimethylchlorosilane). Then the sample was vortexed for 30 seconds, heated at 60 °C for 2 hr and the sample was loaded in split 1:30 on the column Zebron, ZB-5 (L = 30 m, ID = 25 mm, FT = 0.25 μm, Phenomenex, USA). The starting temperature of chromatographic analysis was 100 °C and the temperature increased 15 °C per minute until the temperature reached 320 °C. The column was standardized with sugars, such as α- and β-glucose what allows to indicate the presence such sugars in the analyzed sample.

### Protein folding and aggregation tests using nanoDSF technology (NanoTemper)

The thermal unfolding experiments were carried out using nanoDSF technology (NanoTemper). The Prometheus instrument was used to monitor unfolding-related tryptophan and tyrosine fluorescence at the emission wavelengths of 330 nm and 350 nm, respectively. The thermal unfolding transition midpoint, T_m_ [°C], which is the inflection point of the unfolding curve and the point at which half of the protein population is unfolded, was determined automatically from the derivative of the curve using the PR.ThermControl software^37^. This method circumvents the need to subjectively determine the baseline levels and allows for the determination of a single or multiple unfolding transition midpoints. The PR.ThermControl software automatically calculates the onset of unfolding using the transition midpoint and the slope of the unfolding signal.

The protein aggregation tests were carried out using backreflection technology (NanoTemper) and the Prometheus instrument. It emits near-UV light at a wavelength that is scattered by aggregated proteins, so that that only non-scattered light reaches the detector. The reduction in backreflected light is taken as a direct measure of aggregation, and is plotted as mAU (Attenuation Units) against temperature.

Protein unfolding and aggregation were detected simultaneously. Briefly, 10 µl of TTPAgp11 (0.25 mg/ml, 23.5 µM) was placed in the capillary either alone or mixed with one of the following sugars (0.15 mg/ml): glucose, galactose, maltose, *N*-acetylglucosamine, *N*-acetylgalactosamine, melibiose, α-lactose and β-lactose. The samples were subjected to a temperature ramp of 2 °C/min from 20 °C to 95 °C and fluorescence was constantly monitored. Data were analyzed with the PR.ThermControl and PR.StabilityAnalysis software packages^37^. The profile of the 350/330 nm fluorescence ratio was used to calculate T_m_ [°C]. The first derivative profile showed the onset temperatures of unfolding and aggregation as well as the occurrence of conformational changes in the protein sample. The scattering curve was used to determine the onset temperature of protein aggregation.

## Supplementary information

Supplementary information
