# Supplementary material for: Biochemical features of the novel Tail Tubular Protein A of Yersinia phage phiYeO3-12
Source: Sci Rep. 2020 Mar 6;10:4196. doi: 10.1038/s41598-020-61145-5 (PMC7060351; doi:10.1038/s41598-020-61145-5)
Supplement: Supplementary file 1 — Supplementary information [file 41598_2020_61145_MOESM1_ESM.doc]

**Biochemical features of the novel Tail Tubular Protein A of *Yersinia* phage phiYeO3-12**

Anna Pyra^1^*, Natalia Urbańska^2,3^, Karolina Filik^3^, Katherine Tyrlik^3^ & Ewa Brzozowska^3^**

^1^ University of Wroclaw, Faculty of Chemistry, 14 F. Joliot-Curie St, Wroclaw, 50383, Poland

^2^ University of Wroclaw, Faculty of Biological Sciences, Institute of Experimental Biology, 6 Kanonia St, Wroclaw, 50328, Poland

^3^ Hirszfeld Institute of Immunology and Experimental Therapy, Polish Academy of Sciences, 12 R. Weigl St, Wroclaw, 53114, Poland

* anna.pyra@chem.uni.wroc.pl

** ewa.brzozowska@hirszfeld.pl

BLAST amino acid sequence analysis [20] showing similarity of TTPAgp11 to other TTPs.

| Tail Protein (TP) | Bacteria | % of amino acid sequence identity |
| --- | --- | --- |
| Tail Tubular Protein A (TTPA) | *Yersinia* | 72-100 |
|  | *Citrobacter* | 99-100 |
|  | *E. coli* | 68-100 |
|  | *Enterobacter* | 99 |
|  | *Enterobacteria* | 74-99 |
|  | *Salmonella* | 76-99 |
|  | *Serratia* and *Leclercia* | 98 |
|  | *Pectobacterium, Erwinia* and *Kluyvera* | 72-75 |
|  | *Dickeya* | 68-70 |
|  | *Pseudomonas,* *Pseudobacterium* and *Morganella* | 62-64 |
| Tail Fiber Protein B (TFPB) | *Yersinia*, *E. coli* and *Stenotrophomonas* | 76-81 |

[20] Altschul, S. F., Madden, T. L., Schaffer, A. A., Zhang, J., Zhang, Z., Miller, W., Lipman, D. J., Gapped BLAST and PSI-BLAST: a new generation of protein database search programs. *Nucleic Acids Res.* 25, 3389-3402 (1997).


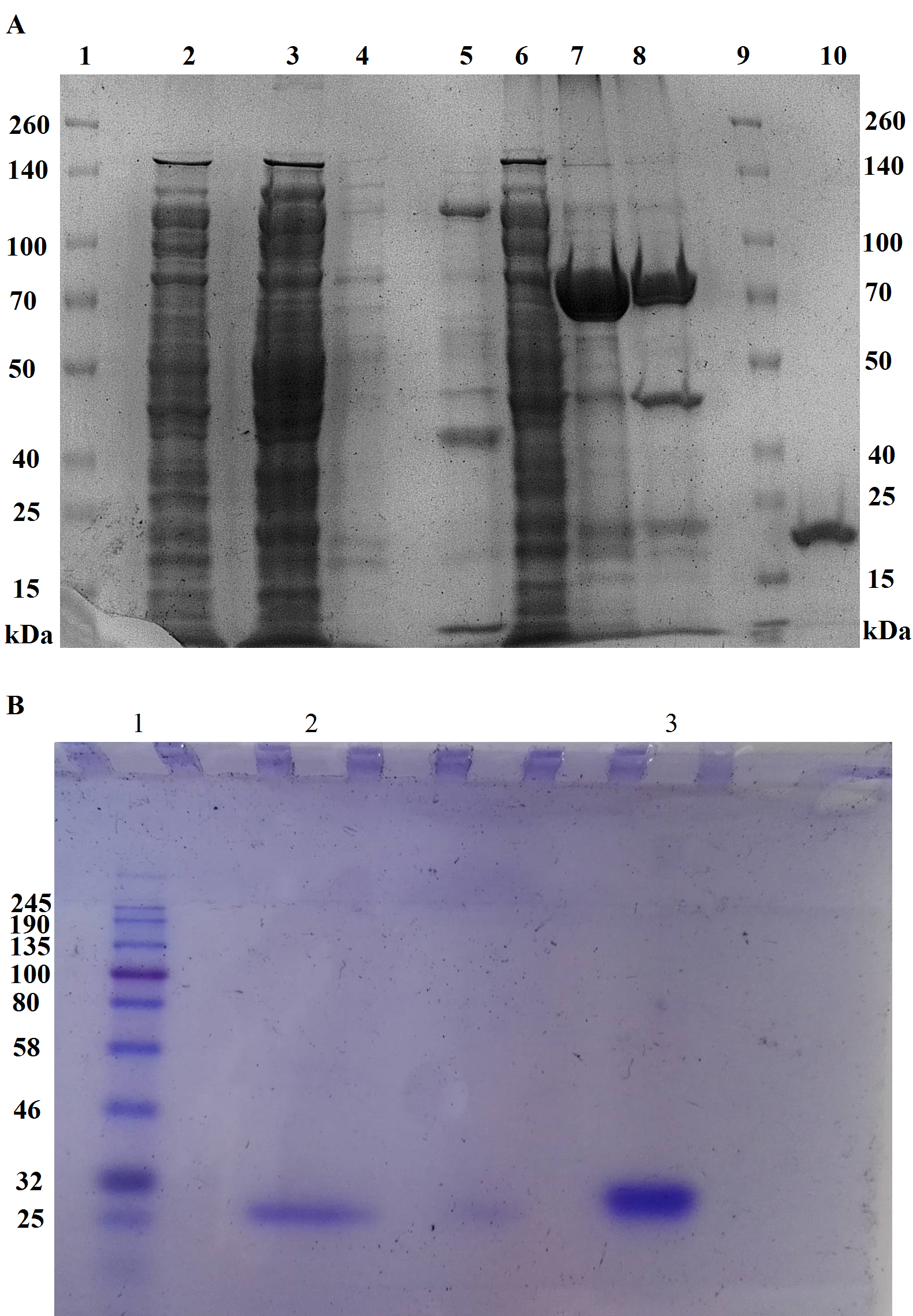


**Figure 3.** Analysis of TTPAgp11 from *Yersinia* phage phiYeO3-12 using 10% (A) and 12.5% (B) SDS-PAGE. (A) The lanes are as follows: (1) Spectra Multicolor Broad Range Protein Ladder (Bio-Rad); (2) the pellet and (3, 4) the supernatant after sonication and centrifugation of *E.coli* BL21 Star(DE3) cells (lane 4 - 15 times less sample volume); (5) the fraction eluted with 20 mM imidazole and (6) the flow through fraction after the first round of nickel-immobilized affinity column; (7) MBP-TTPAgp11 fusion protein after purification by the first round of Ni2+-affinity chromatography; (8) MBP-TTPAgp11 fusion protein after purification by the first round of Ni2+-affinity chromatography and TEV protease cleavage; (9) Spectra Multicolor Broad Range Protein Ladder (Bio-Rad); (10) TEV protease. (B) The lanes are as follows: (1) Color Prestained Protein Standard, Broad Range (Bio-Rad); (2) TTPAgp11 after purification by the second round of Ni2+-affinity chromatography; (3) TEV protease.
